# Neuroinflammatory Mechanisms in Depression: From Biomarkers to Anti-Inflammatory Therapy

**DOI:** 10.3390/brainsci16060632

**Published:** 2026-06-12

**Authors:** Sixian Li, Qixian Wang, Junhua Li, Qi Luo

**Affiliations:** 1 Queen Mary School, Jiangxi Medical College, Nanchang University, Nanchang 330000, China; 13183095688@163.com (S.L.); wang_qixian_2004@163.com (Q.W.); 16688166160@163.com (J.L.); 2 School of Basic Medical Sciences, Jiangxi Medical College, Nanchang University, Nanchang 330000, China

**Keywords:** major depressive disorder (MDD), depression, inflammatory biomarkers, pro-inflammatory cytokines, neuroinflammation, anti-inflammatory therapy

## Abstract

Major depressive disorder (MDD) is a complex and heterogeneous psychiatric disorder with a high prevalence. Neuroinflammation may define biologically distinct patient subgroups with different mechanisms, clinical phenotypes, and treatment responses. This narrative review integrates current evidence around three linked questions: how neuroinflammatory processes contribute to depression, how biomarkers can identify clinically relevant inflammatory phenotypes, and how these findings can inform anti-inflammatory treatment strategies. The major mechanisms discussed include microglial activation and neuroimmune signaling, hypothalamic–pituitary–adrenal axis dysregulation and glucocorticoid receptor resistance, kynurenine pathway alterations, and cytokine-driven impairment of neurogenesis and synaptic plasticity. These pathways interact with stress responses, neurotransmitter systems, and neuronal function, while their expression may vary according to sex, age, hormonal status, disease stage, and treatment exposure. These interconnected pathways may contribute to depressive symptoms by disrupting neurotransmitter systems and impairing neural plasticity. In addition, this review discusses several candidate biomarkers, including C-reactive protein (CRP), interleukin-1β (IL-1β), interleukin-6 (IL-6), tumor necrosis factor-α (TNF-α), brain-derived neurotrophic factor (BDNF) and transforming growth factor-β1 (TGF-β), which may support patient stratification, treatment prediction, and assessment of target engagement. Clinical trials of anti-inflammatory agents have shown inconsistent and generally modest effects in unselected MDD populations. By integrating mechanistic evidence with biomarker-guided therapeutic implications, this review aims to clarify how neuroinflammatory research may inform more precise and individualized treatment strategies for depression.

## 1. Introduction

Major depressive disorder (MDD) is a complex and heterogeneous psychiatric condition characterized by persistent low mood, anhedonia, and cognitive and somatic disturbances that substantially impair daily functioning [1]. Depression affects hundreds of millions of people worldwide and is one of the leading causes of disability. Epidemiological data indicate a high lifetime prevalence. Depressive episodes affect approximately 4.7% of the world’s population over a 12-month period, particularly among vulnerable groups such as older adults and individuals with chronic medical conditions [2]. Beyond its high prevalence, MDD is associated with considerable morbidity and mortality [3].

Despite decades of research, the pathophysiology of MDD remains incompletely understood. Current diagnosis and treatment strategies remain mainly symptom-based, with many patients failing to achieve remission. This highlights the need for mechanism-based biomarkers and novel therapeutic targets. The early explanations of the biological mechanisms of depression were mainly based on the monoamine hypothesis, which proposed that depressive symptoms resulted from insufficient monoamine neurotransmission in the brain [4]. This framework established a neurochemical basis for depression and guided the development of modern antidepressant therapies. However, accumulating evidence suggests that the monoamine hypothesis alone is insufficient to fully explain the pathogenesis of major depressive disorder [5]. These observations suggest that depression involves more complex biological processes, prompting increasing attention to immune and inflammatory mechanisms.

Neuroinflammation is increasingly recognized not only as a response to injury or infection but also as an important contributor to the pathogenesis of neuropsychiatric disorders, including depression [6]. Neuroinflammatory processes have been associated with structural and functional alterations in brain regions involved in mood regulation, particularly the amygdala and hippocampus [7]. Increasing evidence indicates that psychosocial stressors can trigger neuroinflammatory activation, which has been closely associated with the development and progression of depressive symptoms [8]. Persistent neuroinflammatory signaling may contribute to depressive-like behaviors and promote the pathophysiological progression of major depressive disorder [6].

Depression is a highly heterogeneous disease. Patient stratification should further account for biological factors that influence immune responses, including sex, age, and hormonal status, all of which may contribute to variability in the inflammation–depression association. Women have an approximately twofold higher lifetime risk of experiencing a major depressive episode than men [9], and their differences are also observed in symptom presentation, treatment response, and immune system biology.

Reliable biomarkers are needed to identify patients with inflammation-related depression. Biomarkers are measurable biological indicators reflecting pathological processes or treatment responses [10]. While biomarker-guided approaches are widely used in fields such as oncology [11,12], psychiatry still lacks biological markers in clinical practice despite growing evidence that psychiatric disorders involve measurable biological alterations [13,14]. Increased levels of peripheral inflammatory biomarkers are present in patients with depression, and the level of inflammation correlates with the severity of specific symptoms [15]. In the context of depression, biomarkers hold considerable potential for clarifying disease mechanisms, identifying biologically distinct patient subgroups, and predicting treatment response, thereby promoting the development of targeted anti-inflammatory treatment strategies for depression [16]. This framework also helps explain why anti-inflammatory interventions may show benefit in selected inflammatory subgroups but inconsistent efficacy across broad MDD populations.

This review aims to discuss the role of neuroinflammation in the pathophysiology of depression. By exploring the cellular, molecular, and systemic mechanisms of the neuroinflammatory process, particularly the activation of microglial cells, HPA axis dysregulation, kynurenine pathway dysregulation, and cytokine-driven neuroplasticity and neurogenesis impairment in depression, this review discusses how these pathways may contribute to depressive symptoms and disease progression. In addition, this review emphasizes the bidirectional relationship between depression and inflammation and its effects on neurotransmitter systems. The review aims to summarize the application of candidate biomarkers related to neuroinflammation in depression and to provide information for future therapeutic strategies targeting the neuroinflammatory pathways of depression.

## 2. Review Methodology

This article was designed as a narrative review. Relevant literature was selected from PubMed, Web of Science, Scopus, and Google Scholar, with priority given to clinical studies, meta-analyses, translational studies, and mechanistic work addressing neuroinflammation, inflammatory biomarkers, and anti-inflammatory therapy in depression. Search terms included major depressive disorder, depression, neuroinflammation, microglia, cytokines, CRP, IL-6, TNF-α, IL-1β, BDNF, kynurenine pathway, HPA axis, treatment-resistant depression, biomarkers, patient stratification, and anti-inflammatory treatment.

The literature search mainly covered studies published up to 2025, with emphasis on articles from the past decade. Articles were selected according to their relevance to the mechanistic and translational focus of this review. Studies were excluded if they were not directly related to depression, neuroinflammation, inflammatory biomarkers, or anti-inflammatory treatment strategies, or if their clinical or mechanistic relevance to the review topic was limited. Because the aim of this review was to provide an integrative narrative synthesis rather than systematic evidence ranking, formal quality assessment and meta-analysis were not performed.

## 3. Patient Stratification

Before discussing specific neuroinflammatory mechanisms, it is important to consider the biological heterogeneity of MDD. Neuroinflammatory alterations may define biologically enriched subgroups characterized by distinct immune profiles, clinical phenotypes, and treatment-relevant vulnerabilities. Therefore, patient stratification provides a useful framework for interpreting mechanistic findings in neuroinflammation-related MDD. Furthermore, patient stratification is essential for translating neuroinflammatory findings into clinically useful treatment strategies. Current evidence suggests that anti-inflammatory therapies are unlikely to benefit all patients with MDD, but may be more relevant for biologically enriched subgroups characterized by elevated immune activation [17].

Stratification should also consider biological variables that shape immune reactivity. Sex, age, and hormonal status contribute to heterogeneity in the inflammation–depression relationship.

Sex should be incorporated as a key biological variable, as genetic, hormonal, and developmental factors contribute to distinct immune profiles in men and women. Such differences may influence inflammatory responses, cellular repair, and ultimately contribute to sex-specific variation in depressive symptomatology and disease severity [18]. Women generally show stronger innate and adaptive immune responses and may be more vulnerable to inflammation-related depressive phenotypes [19], while hormonal transitions such as the peripartum period and perimenopause may destabilize neuroimmune balance and increase susceptibility to mood dysregulation [20,21].

Aging is another important stratification factor, as immunosenescence and inflammaging are associated with persistent low-grade increases in IL-6, TNF-α, and CRP and may enhance vulnerability to late-life depression [22]. Aging-related immune changes have also been associated with greater depressive burden and stronger reactivity to inflammatory stressors in older adults [23].

However, single peripheral biomarkers are unlikely to capture the full complexity of depression-related neuroinflammation. More refined stratification may require cellular immune phenotyping, neuroimaging evidence of regional neuroinflammation, assessment of BBB dysfunction, and multi-omics signatures integrating immune, metabolic, and genetic data [24,25]. This stratification framework provides the basis for interpreting the major neuroinflammatory mechanisms discussed below. And it also may help clarify the inflammatory characteristics of depression and support the design of clinical trials based on biomarkers.

## 4. Neuroinflammatory Mechanisms in Depression

### 4.1. Microglial Activation and Neuroimmune Signaling

Microglia are the resident immune cells of the central nervous system and play a critical role in maintaining immune surveillance and homeostasis in the brain [26]. Microglia adopt activated phenotypes in response to inflammatory stimuli, infection, or tissue damage. This is followed by proliferation and the production of pro-inflammatory mediators such as IL-1β, TNF-α, and IL-6 [27,28].

**Figure 1:**
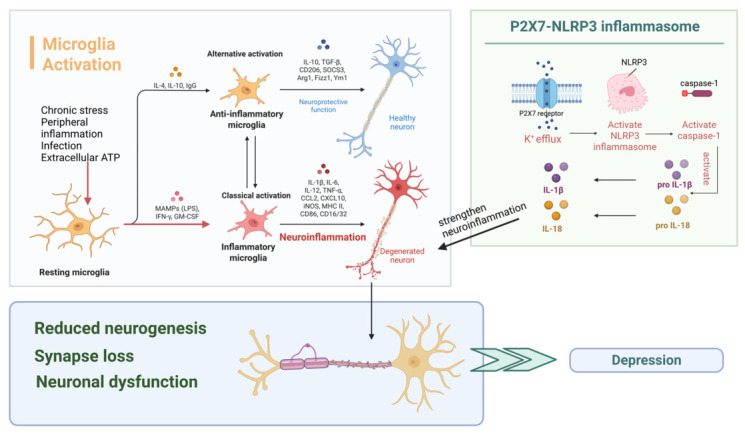
Microglial activation-mediated neuroinflammatory signaling in depression. Created with BioRender.com. This figure summarizes how microglial activation contributes to depression through neuroimmune signaling. Chronic stress, peripheral inflammation, infection-related signals, and extracellular ATP induce the activation of microglia and promote their shift toward a pro-inflammatory phenotype. These states release pro-inflammatory mediators, including IL-1β, IL-6, and TNF-α, which amplify neuroinflammatory signaling and contribute to neuronal injury. In contrast, regulatory or repair-associated microglial states produce anti-inflammatory mediators and support neuronal integrity. Extracellular ATP activates the P2X7 receptor, induces potassium efflux, and promotes activation of the NLRP3 inflammasome. Subsequent caspase-1 activation cleaves pro-IL-1β and pro-IL-18 into their mature forms, thereby reinforcing microglia-mediated neuroinflammation. Persistent inflammatory signaling contributes to reduced neurogenesis, synapse loss, neuronal dysfunction, and impaired neuronal connectivity, which may increase vulnerability to depressive symptoms.

Increasing evidence indicates that microglial activation is closely associated with neuroinflammation in major depressive disorder (MDD) [29] (Figure 1). Numerous investigations have confirmed the presence of neuroinflammation characterized by microglial activation in mood disorders, and this activation appears to vary with disease progression and treatment status [30,31]. The evolution of microglial activation tends to stabilize after antidepressant treatment, while untreated patients with longer courses of depression show higher levels of microglial activation than those with shorter disease durations [32,33].

Converging evidence from neuroimaging and postmortem studies suggests that microglial alterations in major depressive disorder (MDD) are region-specific and state-dependent rather than merely increased or decreased. For instance, some post-mortem studies have reported that patients with MDD did not show a significant difference in the density of microglia compared to the control group [34]. However, in the anterior cingulate cortex of patients with depression who died by suicide, an increase in the ratio of activated microglia to branched microglia has been observed [34]. This suggests that microglial activation state, rather than cell density alone, may be more relevant to depression-related neuroinflammation.

Positron emission tomography (PET) studies using translocator protein (TSPO) as a marker of microglial activity have consistently demonstrated elevated signals in key mood-related regions, including the prefrontal cortex (PFC) and anterior cingulate cortex (ACC), particularly during acute depressive episodes [32,35]. Notably, TSPO binding in the ACC has been shown to correlate with the severity of depressive symptoms, and longitudinal imaging further indicates that microglial activity is associated with illness duration and treatment exposure [36].

Microglia exhibit remarkable phenotypic plasticity and can polarize into different functional states [37]. The pro-inflammatory phenotype, typically induced by stimuli such as lipopolysaccharide (LPS) or interferon-γ, releases inflammatory mediators including IL-1β, IL-6, and TNF-α, thereby amplifying neuroinflammatory responses [38,39]. In contrast, the anti-inflammatory phenotype, induced by cytokines such as IL-4 or IL-13, is associated with anti-inflammatory functions and tissue repair [38,39]. Microglial phenotypic changes in vivo are highly dynamic and context-dependent, involving not only the loss of homeostatic functions but also the acquisition of neurotoxic or repair-associated properties that may vary according to the stage and intensity of neuroinflammation [40]. Therefore, a simple binary M1/M2 classification cannot fully capture the diverse and overlapping microglial states observed under pathological conditions. In this review, the M1/M2 terminology is used only as a simplified descriptive framework, while microglial activation is better understood as a continuum of functional states rather than two fixed and mutually exclusive phenotypes.

Psychological stress represents a major trigger for microglial activation. Experimental studies have shown that chronic stress induces a shift toward the pro-inflammatory microglial phenotype, which promotes neuroinflammation by recruiting peripheral immune cells and activating astrocytes [41,42]. Activated microglia produce signaling molecules that modify astrocytic activity and enhance inflammatory signals in the central nervous system, aggravating neuroinflammatory processes [43]. The inflammatory environment can inhibit neurogenesis in the dentate gyrus of the hippocampus and disrupt synaptic plasticity, ultimately leading to depressive-like behaviors [44]. Activated microglia not only release inflammatory mediators but also control synaptic pruning, which controls synaptic remodeling. Excessive pruning under neuroinflammatory settings can result in synapse loss and poor neuronal connection, which may contribute to cognitive impairment and symptoms of depressive disorders, despite the fact that this process is necessary for normal neural circuit growth [45,46]. Persistent neuroinflammation may enhance signaling between the peripheral immune system and the brain, further amplifying inflammatory responses [44].

The P2X7–NLRP3 inflammasome signaling pathway has been identified as a key molecular mechanism underlying microglia-mediated neuroinflammation [5]. The activation of the purinergic P2X7 receptor by extracellular ATP induces potassium efflux and facilitates the activation of the NLRP3 inflammasome in microglia [47]. This process subsequently activates caspase-1, leading to the cleavage of pro-IL-1β and pro-IL-18 into their biologically active forms [48]. Overproduction of IL-1β can disrupt synaptic transmission and trigger excitotoxic neuronal injury [49]. Genetic variants of the P2X7 receptor are linked to elevated susceptibility to depression, and experimental research indicates that the deletion of the P2X7 receptor can inhibit LPS-induced depressive-like behavior in animal models [50]. These findings highlight the P2X7–NLRP3–IL-1β axis as a critical link between peripheral immune activation and central neuroinflammatory processes.

Activated microglia can also influence neurotransmission through additional mechanisms. In addition to releasing inflammatory mediators, microglia produce glutamate and metabolize kynurenine into quinolinic acid, a neurotoxic metabolite that can enhance glutamatergic neurotransmission and contribute to excitotoxicity [51]. Together with impaired astrocytic glutamate uptake, these changes may further disrupt neuronal signaling and promote the development of depressive symptoms [52].

Sex differences may also shape neuroinflammatory mechanisms at the level of innate immune sensing. Because the regulatory effects of gender-related pattern recognition receptors may lead to the activation of the neuroimmune system [53], this explains the gender-specific differences in neuroinflammation in depression. For example, toll-like receptor 4 (TLR4), a pattern-recognition receptor that detects danger-associated molecular patterns, can trigger pro-inflammatory signaling in peripheral innate immune cells and microglia. Experimental evidence suggests that TLR4 expression and activity are modulated by sex hormones. Testosterone exposure reduced TLR4 expression in cultured macrophages [53]. In the central nervous system, TLR4 signaling contributes to microglia-mediated inflammatory responses [54]. These findings suggest that sex hormone-dependent modulation of innate immune signaling may partly contribute to sex-related differences in inflammation-associated depressive vulnerability.

### 4.2. Hypothalamic–Pituitary–Adrenal (HPA) Axis Dysregulation

**Figure 2:**
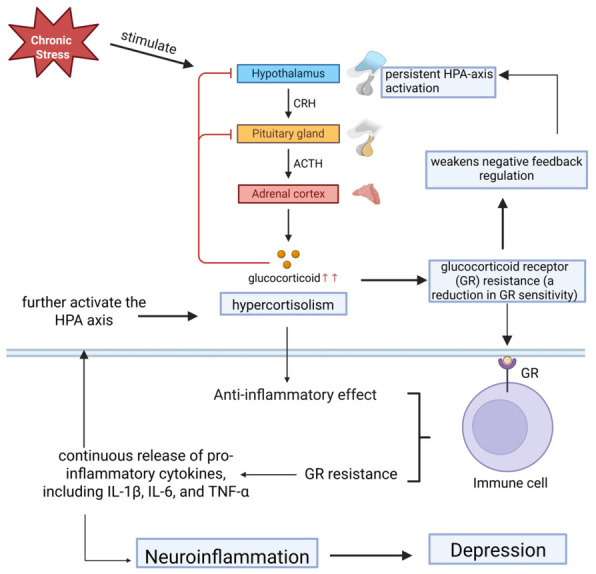
Bidirectional interaction between neuroinflammation and HPA axis dysregulation in depression. Created with BioRender.com. Chronic stress activates the hypothalamic–pituitary–adrenal (HPA) axis, promoting the sequential release of corticotropin-releasing hormone (CRH), adrenocorticotropic hormone (ACTH), and glucocorticoids from the hypothalamus, pituitary gland, and adrenal cortex, respectively. Under physiological conditions, glucocorticoids activate glucocorticoid receptors (GRs) to suppress inflammatory responses and provide negative feedback at the hypothalamus and pituitary, thereby maintaining HPA-axis homeostasis. In a subset of patients with major depressive disorder, reduced GR sensitivity or GR resistance weakens both of these regulatory functions. Impaired negative feedback contributes to persistent HPA-axis activation and hypercortisolism, whereas diminished GR-mediated immunosuppression permits immune cells to sustain the release of pro-inflammatory cytokines, including IL-1β, IL-6, and TNF-α. These cytokines can further activate the HPA axis and impair GR signaling, establishing a self-reinforcing cycle between stress-system dysregulation and inflammation. Persistent cytokine signaling and neuroinflammation may subsequently disrupt neuronal and hippocampal function, thereby contributing to depressive symptoms.

The hypothalamic–pituitary–adrenal (HPA) axis regulates stress responses and has been consistently implicated in major depressive disorder [42] (Figure 2). Corticotropin-releasing hormone (CRH) released by paraventricular neurons in the hypothalamus induces secretion of adrenocorticotropic hormone (ACTH) of the pituitary, which subsequently stimulates release of glucocorticoids of the adrenal glands [55]. Glucocorticoids are known to affect nearly every organ system. They primarily function by activating glucocorticoid receptors (GRs), which produces anti-inflammatory and immunosuppressive effects while also modulating neuronal activity in the hippocampus [56,57].

Under physiological conditions, glucocorticoids inhibit their own synthesis through GR-dependent negative feedback at the hypothalamus and pituitary, maintaining HPA axis homeostasis [58]. The dysregulation and imbalance of the HPA axis are present in a substantial subset of patients with MDD, which is caused by decreased GR-mediated negative feedback within the HPA axis, also known as glucocorticoid receptor (GR) resistance [59]. GR resistance prevents glucocorticoids from exerting their anti-inflammatory effects and leads to increased release of pro-inflammatory cytokines [60]. Moreover, it disrupts glucocorticoid-negative feedback, leading to persistent HPA axis activation and glucocorticoid overproduction [60].

Several inflammatory signaling mediators, including NF-κB, p38 MAPK, and STAT5, have been linked to cytokine-induced suppression of GR function [61]. For example, pro-inflammatory cytokines have been shown to prevent GR translocation from the cytoplasm to the nucleus via STAT5 phosphorylation [62]. IL-1α reduces GR nuclear translocation and attenuates dexamethasone-induced GR gene activity via p38 MAPK activation [63]. In addition, TNF-α suppresses GR function through p38 and JNK pathways [64], as well as through protein–protein interactions that disrupt GR-dependent transcriptional control [65]. Anti-inflammatory modulation may partially restore impaired GR function [66].

Simultaneously, immune cells with GR resistance produce more pro-inflammatory cytokines because the normal immunosuppressive effects of glucocorticoids are reduced [67]. In addition, glucocorticoids have been reported to enhance NLRP3 expression, thereby sensitizing inflammasome-dependent release of IL-1β, TNF-α, and IL-6, and act synergistically with TNF-α to increase expression of pro-inflammatory genes such as serpinA3 [68]. This may promote a pro-inflammatory environment, worsen neuroinflammatory signaling, and contribute to the pathophysiology of depression [69]. Therefore, the interaction is bidirectional.

Biological sex may further shape HPA-axis dysregulation in depression. Studies have shown that there are differences in the response to cortisol between men and women, but the direction of these differences may depend on the developmental stage and the type of stressor [70,71]. Female sex hormones may influence the regulatory mechanism of the HPA axis by acting on corticosteroid receptors in brain regions related to stress such as the hypothalamus and amygdala, as well as by regulating neurotransmitter systems involved in the control of the HPA axis [72]. Estrogen has complex, receptor- and region-specific effects on glucocorticoid signaling, whereas progesterone may attenuate HPA-axis-negative feedback through interactions with corticosteroid receptors [73,74]. These findings suggest that HPA-axis abnormalities in MDD should be interpreted in relation to sex, developmental stage and hormonal status across all patients.

### 4.3. Kynurenine Pathway Dysregulation

**Figure 3:**
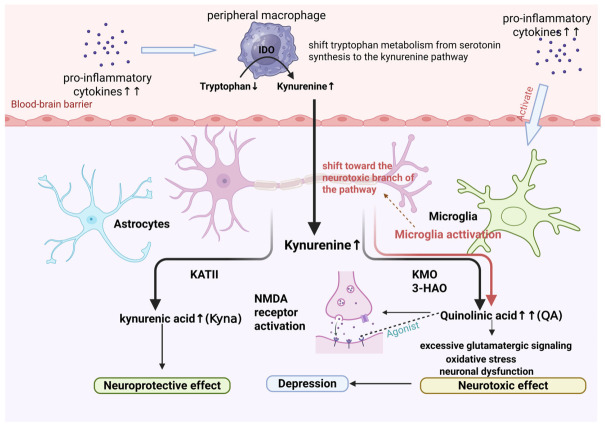
The role of kynurenine pathway dysregulation in depression. Created with BioRender.com. The figure summarizes the role of kynurenine pathway dysregulation in depression. Inflammatory signals activate IDO and TDO, shifting tryptophan metabolism from serotonin synthesis to the kynurenine pathway. This leads to increased kynurenine production. After crossing the blood–brain barrier, kynurenine is metabolized into either kynurenic acid (Kyna), a neuroprotective metabolite mainly produced by astrocytes, or quinolinic acid (QA), a neurotoxic metabolite preferentially generated by microglia and macrophages. In depression-related inflammatory conditions, this balance is shifted toward the QA branch, resulting in NMDA receptor overactivation, excitotoxicity, oxidative stress, and neuronal dysfunction. These alterations impair neurogenesis and synaptic plasticity and ultimately contribute to depressive symptoms.

The kynurenine (Kyn) pathway is the principal metabolic route of tryptophan and is recognized as a key mechanism linking neuroinflammation and depression [75] (Figure 3). Under physiological conditions, tryptophan metabolism is balanced between serotonin synthesis and degradation through the kynurenine pathway [76]. However, increasing evidence indicates that inflammatory activities can disrupt the balance and change the metabolism of tryptophan, which may affect mood regulation, neurotransmission, and neuronal survival [77].

Under inflammatory conditions, pro-inflammatory cytokines such as interleukin-1β, interleukin-6, and tumor necrosis factor-α can activate the enzymes indoleamine-2,3-dioxygenase (IDO) and tryptophan-2,3-dioxygenase (TDO) [78,79]. Because IDO and TDO catalyze the initial step of this pathway, their activation substantially increases the conversion of tryptophan into kynurenine while reducing tryptophan availability for serotonin synthesis [80].

The resulting increase in kynurenine leads to the accumulation of neuroactive metabolites that can significantly influence neuronal function [81]. Kynurenine can cross the blood–brain barrier and is further metabolized within the central nervous system through two major branches [82]. Astrocytes primarily convert kynurenine into kynurenic acid (Kyna), a neuroprotective metabolite, while microglia and macrophages preferentially metabolize kynurenine into neurotoxic compounds such as quinolinic acid (QA) [82]. Under normal conditions, the balance between these neuroprotective and neurotoxic metabolites contributes to neuronal homeostasis [76]. In depression, microglia activation can shift this metabolic balance toward the neurotoxic branch of the pathway, resulting in increased production of QA [83]. QA is a potent agonist of *N-methyl-D-aspartate (NMDA)* receptors and can induce excessive glutamatergic signaling, oxidative stress, and neuronal dysfunction [84]. These pathological processes can disrupt neural circuits involved in mood regulation, particularly in brain regions such as the hippocampus and prefrontal cortex [85].

An imbalance between neuroprotective and neurotoxic metabolites has also been associated with structural and functional brain alterations observed in major depressive disorder [79]. Dysregulation of the kynurenine pathway has been linked to reduced hippocampal neurogenesis and impaired synaptic plasticity, both of which are frequently found in depression [86]. Moreover, alterations in kynurenine metabolism may interact with other biological pathways involved in depression, including microglial activation and stress-related neuroendocrine responses [76].

Clinical findings on kynurenine metabolites in depression are not fully consistent. Although inflammatory activation is often proposed to drive tryptophan metabolism toward the kynurenine pathway and its neurotoxic branch, empirical findings vary across brain regions, sample types, and clinical states. Postmortem studies have reported increased microglial quinolinic acid expression in the subgenual and supracallosal anterior cingulate cortex of patients with MDD, whereas reduced quinolinic acid has been observed in selected hippocampal subfields in unipolar and bipolar depression [87]. Likewise, meta-analytic evidence indicates that in patients with depression, the overall levels of kynurenine and kynurenic acid have decreased, while in patients not using antidepressants, the level of quinolinic acid has increased [87,88]. These conflicting research results indicate that the dysregulation of the tryptophan pathway may be region-specific and state-dependent, rather than simply a general increase in pathway activity. Differences in inflammatory status, antidepressant exposure, illness stage, peripheral versus central sampling, and the relative activity of astrocytic versus microglial metabolic branches may all influence whether the pathway shifts toward neuroprotective or neurotoxic metabolites.

Clinical and experimental findings further support the role of the kynurenine pathway in depression [89,90,91,92]. For instance, patients receiving interferon-α therapy have lower tryptophan levels, higher kynurenine concentrations, and elevated Kyn/Trp ratios, which can lead to depression symptoms [92]. Similarly, inflammatory stimulation in animal models has been found to increase kynurenine pathway activity and induce depressive-like behaviors [93]. These findings highlight the kynurenine pathway as an important link between immunological activation, neurotransmitter imbalance, and the pathophysiology of depression.

### 4.4. Cytokine-Driven Neuroplasticity and Neurogenesis Impairment

Neurogenesis refers to the generation of new neurons from neural stem cells, a multistep process that includes stem cell proliferation, neuronal differentiation, and the eventual integration of newly formed neurons into existing neural circuits [94,95]. Among psychiatric disorders, major depressive disorder (MDD) has been the most extensively investigated in relation to adult neurogenesis [96]. Accumulating evidence suggests that neurogenesis is closely linked to neuroinflammation and the activity of pro-inflammatory cytokines [97].

This association is biologically plausible because receptors for pro-inflammatory cytokines are highly concentrated in cognition-related brain regions, particularly the hippocampus [98,99]. Multiple studies have shown that pro-inflammatory cytokines have adverse effects on neurogenesis in the adult hippocampus. Experimental work has shown that pro-inflammatory cytokines such as IL-6, together with microglial activation, suppress hippocampal neurogenesis, while neutralization of IL-6 can restore impaired neurogenic activity, supporting a central role for IL-6 in this process [100]. Consistently, NF-κB-mediated IL-6 signaling has also been linked to depression-like behaviors and reduced proliferation of hippocampal cells in animal studies [101,102]. TNF-α has also been implicated in impaired neurogenesis, but its effects appear to be receptor-dependent rather than uniformly deleterious. Previous studies suggest that TNFR1, which contains an intracellular death domain, is more closely associated with suppression of hippocampal neurogenesis, whereas TNFR2 may exert more supportive or regenerative effects [103,104,105]. This receptor-specific distinction indicates that cytokine-driven neuroplastic changes in depression cannot be reduced to a simple uniformly harmful inflammatory model. IL-1β has also been linked to impaired neurogenesis, particularly in the hippocampus [106,107]. It contributes to interferon-γ-induced suppression of neurogenesis [108], and inhibition of IL-1β has been shown to prevent the reduction in neurogenesis induced by acute stress and anti-neurogenic effects of chronic stress [109]. Notably, kynurenine 3-monooxygenase inhibition has been reported to reverse IL-1β-induced neurogenesis impairment, suggesting that IL-1β may disrupt hippocampal neurogenesis at least partly through kynurenine pathway activation [110].

More broadly, depression is increasingly understood as a disorder of impaired neuroplasticity rather than simply reduced neurogenesis alone [111]. In addition to neuronal renewal, neuroplasticity includes synaptogenesis, dendritic remodeling, and changes in synaptic strength, all of which are essential for cognitive flexibility and emotional adaptation [112]. Deficits in these processes have been reported in key mood-related regions, particularly the hippocampus and prefrontal cortex, and may be more directly relevant to depressive pathology than neurogenesis alone [112,113,114].

## 5. Major Neuroinflammatory Biomarkers in Depression

The role of inflammatory mechanisms in the pathophysiology of depression is receiving increasing attention, which has also drawn widespread interest in immunotherapy approaches and biomarker-guided treatments [115]. Accumulating evidence indicates that pro-inflammatory markers including C-reactive protein (CRP), interleukin-6 (IL-6), and tumor necrosis factor-α (TNF-α) are implicated in the pathophysiology of major depressive disorder (MDD) [116]. An increasing number of research studies have shown that the levels of inflammatory markers in patients with depression are significantly higher than those in the healthy control group (Table 1) [117]. More importantly, the levels of cytokines may have clinical translational value and can serve as important indicators for treatment stratification [117]. Studies suggest that appropriately modulating these inflammatory pathways may alleviate depressive symptoms [116].

### 5.1. Pro-Inflammatory Cytokine Markers

#### 5.1.1. Interleukin-1β (IL-1β)

Interleukin-1β (IL-1β) is a pro-inflammatory cytokine initially implicated in depression, often discussed alongside IL-6 and TNF-α [130]. Danger-associated signals can activate inflammatory pathways via inflammasomes or toll-like receptors (TLRs), which in turn promote increases in IL-1β [131,132]. Untreated individuals with depression have been reported to show elevated IL-1β levels in cerebrospinal fluid (CSF). Clinically, IL-1β has been associated with phenotype severity in specific populations. In adults older than 60 years, IL-1β levels were reported to scale with illness severity [133]. Increased IL-1β has also been reported in postpartum depression [134]. Endogenous IL-1 receptor antagonist (IL-1RA) can reduce inflammation caused by IL-1β and has been shown to be neuroprotective. Accordingly, elevated IL-1RA has been linked to better treatment outcomes, which supports the possibility of using it as a marker for antidepressant response [135].

#### 5.1.2. Tumor Necrosis Factor-α (TNF-α)

Tumor necrosis factor-α (TNF-α) is a multifunctional pro-inflammatory cytokine belonging to the TNF superfamily [136]. TNF-α levels in the peripheral blood have been studied as a clinical biomarker to predict disease severity and treatment outcomes. Das et al. reported that TNF was elevated in MDD and higher TNF levels tracked with greater symptom severity [137]. Although TNF-α levels were higher in individuals with depression than in controls overall, there is no significant difference in this correlation in terms of gender [138].

TNF-related signaling is frequently coupled with NF-κB, a transcriptional pathway linked to depression-related neuroinflammation [139]. NF-κB has been reported to be expressed in multiple immune cell types and at the blood–brain barrier (BBB), consistent with a role in immune-to-brain communication [139]. Stress has been described as an early trigger of NF-κB activation [140]. Experimental evidence suggests that inhibiting NF-κB might reduce cerebral inflammatory responses [140]. Glucocorticoids are also reported to regulate this pathway by suppressing NF-κB activity, thereby reducing downstream pro-inflammatory cytokine activation [141]. NF-κB signaling has been linked to stress-induced neurogenesis decreases and depressive symptoms [106].

TNF-α may also have clinical value for treatment matching. Infliximab was found to be beneficial for TRD patients with elevated CRP and TNF-α, suggesting a biomarker-informed strategy to immunomodulatory treatment [142].

#### 5.1.3. Interleukin-6 (IL-6)

Interleukin-6 (IL-6) is produced by multiple cell types, including microglia and astrocytes, in response to inflammatory stimuli. Elevated IL-6 levels have been consistently reported in patients with depression [143]. Furthermore, IL-6 has been identified as a potential predictor of symptom severity. Peripheral IL-6 levels may increase during periods of stress and it will decrease after the stress is relieved [144]. This change is related to the differences in the susceptibility of an individual to depression after experiencing stress [144].

IL-6 may induce depression via multiple neurobiological pathways. It can alter the hypothalamic–pituitary–adrenal (HPA) axis, thereby influencing the regulation of cortisol and the sensitivity to stress [145]. Furthermore, it has been reported that IL-6 is associated with the alteration of neurotransmitter systems, including the impact on serotonin receptor expression, which may affect mood and behavior [146,147]. Experimental studies further indicate that IL-6 would inhibit the process of hippocampal neurogenesis [146], while the use of IL-6 antibodies could restore the impaired neurogenesis response [100]. These findings support the role of IL-6 in linking neuroinflammation with neuroplasticity changes and depressive-like phenotypes. In terms of biological sex, the changes in IL-6 were different. IL-6 was significantly elevated in women with depression relative to female controls, but not in men [138].

IL-6 may also have relevance as a biomarker for therapeutic response. Several studies have reported that IL-6 levels decrease following successful antidepressant treatment, suggesting that part of the therapeutic effect of antidepressants may involve immune modulation [148]. Nevertheless, treatment-related changes in IL-6 are not entirely consistent across modalities. For example, electroconvulsive therapy has been reported to increase IL-6 levels [149], and meta-analytic findings suggest that higher baseline IL-6 may be associated with greater symptom improvement after antidepressant treatment [150]. Another meta-analysis reported that IL-6 levels decreased during antidepressant treatment regardless of clinical outcome [151]. These findings indicate that IL-6 dynamics may depend on baseline inflammatory status, treatment type, sampling time, and clinical subtype, rather than reflecting a simple marker of improvement.

IL-6 acts through both classical signaling and trans-signaling pathways. Trans-signaling involves soluble IL-6 receptors (sIL-6R) and is more closely associated with pro-inflammatory activity [152]. By contrast, classical IL-6 signaling may exert anti-inflammatory effects [153]. Therefore, elevated sIL-6R levels together with increased IL-6 concentrations may indicate enhanced IL-6 trans-signaling and greater inflammatory activation [154,155]. And enhanced IL-6 trans-signaling has been described in acute depressive episodes, including melancholic or atypical depression, as well as in TRD and melancholia [155,156].

### 5.2. Systemic Stratification Markers

#### C-Reactive Protein (CRP)

C-reactive protein (CRP) is a non-specific acute-phase protein that rises in response to systemic inflammation. Evidence indicates that elevated CRP is consistently associated with a higher risk of depressive symptoms [157]. CRP may help predict antidepressant treatment response, and elevated CRP may derive greater benefit from anti-inflammatory interventions [158]. However, CRP has mainly been explored as a stratification marker for anti-inflammatory therapies rather than a direct therapeutic target in depression [159]. Sex-specific findings have also been reported. Compared with healthy controls, women with depression showed significantly higher CRP levels, with a small effect size, whereas no comparable increase was observed in men [138].

CRP may also contribute to biomarker-informed antidepressant selection. In the GENDEP trial, baseline CRP differentially predicted response to escitalopram versus nortriptyline [160]. Patients with lower CRP levels improved more with escitalopram, whereas those with higher CRP levels showed greater improvement with nortriptyline [160]. The result suggests that CRP may have predictive value for treatment stratification rather than merely reflecting systemic inflammation.

### 5.3. Neuroplasticity-Related and Counter-Regulatory Markers

#### 5.3.1. Brain-Derived Neurotrophic Factor (BDNF)

Brain-derived neurotrophic factor (BDNF) is considered to be one of the most prominent and extensively distributed neurotrophic mediators in the nervous system [161,162]. Substantial evidence shows that BDNF promotes neuronal survival and neurogenesis, particularly within the human hippocampal region. Because neuroinflammation disrupts several BDNF-related signaling pathways, reduced BDNF levels in the diseased brain may partially indicate a chronic inflammatory state [163,164]. The kynurenine pathway activation has been shown to be closely related to the downregulation of BDNF synthesis, which may damage the structural integrity of neurons and the process of neurogenesis [145].

Meta-analytic evidence indicates that baseline BDNF levels are reduced in patients with MDD compared with healthy controls [165,166]. Significant reductions in serum BDNF have also been reported in both animal models of MDD and in patients, supporting its use as a candidate biomarker for depression assessment in some studies [167,168]. Lower BDNF levels have also been found in treatment-resistant depression (TRD), especially in the hippocampus [169]. Meanwhile, pharmacotherapy and electroconvulsive treatment may gradually increase serum BDNF, whereas clinical antidepressant effects can emerge more rapidly [170]. These findings suggest that BDNF may reflect altered neuroplasticity in depression, but its relationship with clinical phenotype is not entirely linear.

BDNF alterations are not specific to MDD. Changes in BDNF have also been reported in bipolar disorder, schizophrenia, and neurodegenerative conditions [166,171]. Peripheral BDNF is not sufficient to measure MDD severity and does not reliably discriminate MDD from bipolar disorder or schizophrenia [172]. Moreover, peripheral BDNF levels may be influenced by smoking, diabetes, metabolic status, and other systemic factors that are themselves associated with depression risk [173]. Another limitation is that BDNF signaling is region-specific. Hippocampal BDNF is generally associated with antidepressant-like effects, whereas BDNF activity in the ventral tegmental area and nucleus accumbens may contribute to depression-like effects [174]. Thus, serum BDNF should not be interpreted as a direct indicator for brain-region-specific BDNF signaling.

#### 5.3.2. Transforming Growth Factor-β (TGF-β)

Transforming growth factor-β (TGF-β) is a multifunctional cytokine with important immunoregulatory and anti-inflammatory properties [175]. Emerging evidence suggests that patients with MDD tend to exhibit reduced TGF-β levels, which may contribute to persistent neuroinflammation and mood dysregulation [175]. In addition, lower TGF-β levels have been associated with increased suicide risk, supporting its potential value as a prognostic biomarker [176]. Beyond its anti-inflammatory role, TGF-β signaling is also involved in the regulation of stress responses and has been implicated in the development of mood disorders [175]. Experimental evidence further indicates that imbalance between TGF-β and pro-inflammatory cytokines, together with altered Th17/Treg homeostasis, may promote chronic stress-induced depressive phenotypes [177].

Altered TGF-β1 signaling has been reported in animal models of depression accompanied by cognitive dysfunction [178]. Consistently, reduced plasma TGF-β1 levels have been observed in patients with depression and were associated with greater symptom severity and treatment resistance [179]. More recent human studies have further supported the involvement of TGF-β1 signaling in the pathophysiology of depression [180].

Recent preclinical evidence further suggests that the role of TGF-β1 in stress-related depressive phenotypes may be sex-dependent. In a prenatal stress (PNS) model of adolescent depression, only female PNS-exposed rats exhibited depressive-like behavior in the forced swim test, whereas both sexes showed recognition memory deficits in the novel object recognition test [181]. This pattern indicates that early-life stress may produce distinct behavioral outcomes across sexes. Depressive-like vulnerability was more prominent in females in this model. Importantly, hippocampal TGFβ-R2 expression was increased in resilient PNS rats of both sexes, suggesting that activation of the TGF-β1/TGFβ-R2 pathway may be associated with stress resilience [181]. In contrast, plasma TGF-β1 levels were elevated in male but not female PNS rats, indicating a sex-specific peripheral response that may not directly parallel central vulnerability [181]. These research results indicate that the role of TGF-β1 in depression shows gender differences, and the TGF-β1/TGFβ-R2 pathway may become a drug target for preventing depression.

## 6. Biomarker-Guided Anti-Inflammatory Treatment in Depression

### 6.1. Distinguishing Peripheral Inflammatory Biomarkers from Central Neuroinflammatory Processes

Direct assessment of neuroinflammatory processes in the central nervous system is challenging in humans. Brain tissue biopsy is highly invasive and is rarely performed except when clinically necessary. Although CSF is closer to the CNS, it requires lumbar puncture and is not routinely included in the clinical evaluation of psychiatric disorders [154,182]. As a result, peripheral inflammatory markers in blood are frequently used as more accessible indicators in depression research. However, peripheral inflammatory changes should not be interpreted as direct evidence of central neuroinflammation. The extent to which blood-based markers reflect inflammatory activity in CSF or brain tissue remains uncertain. CRP appears to show consistent evidence as an indicator of inflammation in both peripheral and CNS compartments, but it remains a nonspecific marker of systemic inflammatory activity [159].

The relationship between peripheral inflammation and central neuroinflammatory processes is mediated by regulated interfaces between the blood and CNS. The blood–brain barrier restricts the entry of peripheral inflammatory proteins and immune cells into the CNS under physiological conditions [159,183]. However, pathological conditions may increase BBB permeability [184], allowing cytokines and immune cells to enter the CNS, activate glial cells, and induce central inflammatory responses [185]. In addition to the BBB, the choroid plexus and blood–CSF barrier provide another route for blood–CSF communication, allowing regulated passage of small proteins, including cytokines and chemokines [186,187]. The choroid plexus epithelium also participates in neuroimmune regulation and may recruit inflammatory proteins generated within the CNS into the CSF [188]. Finally, CSF reabsorption into peripheral blood at the arachnoid granulations may allow inflammatory proteins and biochemical waste products from the CNS to enter the circulation [187]. Therefore, peripheral biomarkers may provide clinically useful information for patient stratification and treatment monitoring, but they should be interpreted as indirect indicators rather than direct measures of central neuroinflammation.

### 6.2. Anti-Inflammatory Agents

The effectiveness of antidepressant treatment may be affected by the presence of inflammatory dysregulation in depressed patients. This has led to growing interest in the use of anti-inflammatory agents as adjunctive treatments, particularly in patients with elevated inflammatory burden. In parallel, conventional antidepressants may themselves partly interact with immune pathways by modulating inflammatory mediators and glial signaling [189]. Anti-inflammatory agents explored in depression include NSAIDs, cytokine antagonists, immunomodulatory antibiotics, and agents with secondary anti-inflammatory effects.

#### 6.2.1. NSAIDs

Celecoxib, a selective COX-2 inhibitor, is among the most extensively studied anti-inflammatory agents in depression [190]. In the chronic inflammation-induced depression model of male mice, celecoxib can prevent depressive-like behaviors [190]. The combined treatment with amphetamine and celecoxib reduced the activity of IL-1β and COX-2 in the brain, but did not increase the level of BDNF [190]. Clinical studies further support its role as an adjunctive treatment, with previous trials and meta-analyses indicating that the adjunctive use of celecoxib can enhance antidepressant efficacy [191,192]. Its therapeutic effects may be partly mediated by lowering inflammatory markers such as IL-6 and CRP [193].

Aspirin, a non-selective COX inhibitor, has been explored as a potential adjunctive anti-inflammatory treatment for depression [194]. In the chronic mild stress-induced depression model of male rats, aspirin treatment improved various depressive-like behaviors and biochemical indicators [194]. These findings suggest that aspirin may have an antidepressant effect. However, since this evidence comes from a preclinical model consisting only of male animals, its clinical relevance and general applicability to patients with severe depression remain unclear. In a large placebo-controlled trial of healthy older adults, daily low-dose aspirin did not reduce the incidence of depressive symptoms over a median follow-up of 4.7 years [195]. This negative result suggests that anti-inflammatory approaches may have limited value in unselected populations.

#### 6.2.2. Cytokine Antagonists

Infliximab, a TNF-α antagonist, is one of the clearest examples of a biomarker-informed anti-inflammatory strategy in depression. In a double-blind, placebo-controlled trial of 60 patients with treatment-resistant depression, infliximab did not produce a significant overall antidepressant effect compared with placebo [119]. However, treatment response varied according to baseline inflammatory status. Patients with hs-CRP levels above 5 mg/L showed greater symptom improvement with infliximab, whereas those with lower hs-CRP levels did not appear to benefit [119].

Etanercept, a TNF-α antagonist, has shown potential antidepressant effects in preclinical models. Long-term use of the TNF-α inhibitor etanercept can alleviate anxiety-like behaviors in male rats, suggesting that the TNF-α signaling pathway may be involved in the regulation of emotional behaviors and have similar antidepressant effects [196]. However, since this study did not use a validated depression model, the clinical significance of these results remains unclear [196]. Furthermore, in the rat model of depression induced by cortisol, administration of the TNF-α inhibitor etanercept at the periphery was able to reverse the similar depressive behaviors and memory impairments [197]. However, current evidence is still indirect, as most human data come from populations with comorbid inflammatory illnesses rather than primary depressive disorders [197]. Therefore, etanercept is best considered supportive evidence for the antidepressant relevance of TNF-α inhibition rather than a validated treatment strategy for depression itself.

### 6.3. Evaluation of Anti-Inflammatory Treatment for Depression

Although substantial mechanistic evidence suggests that inflammation is associated with depression, the results of clinical trials of anti-inflammatory drugs conducted on patients with depression have shown only modest improvements and are generally limited. These results may partly reflect the limitations of traditional trial design. Many studies adopted a “one-size-fits-all” approach, including heterogeneous patient populations biologically, without confirming the presence of inflammatory activation, and relying on non-specific outcome indicators such as the overall severity of depression measured by the Hamilton Depression Rating Scale [195,198]. Furthermore, the involvement of targeted mechanisms is rarely evaluated, making it difficult to determine whether the negative outcome is due to the failure of the therapeutic hypothesis or to insufficient regulation of the expected inflammatory pathways [198].

### 6.4. Future Research and Clinical Trial Design Suggestions

Future clinical trials of anti-inflammatory treatments for MDD should integrate patient enrichment, outcome alignment, and assessment of target engagement. First, enrichment strategies should use validated predictive biomarkers to identify patients with a probable inflammatory subtype who are more likely to respond to a specific immune-targeted intervention. For this purpose, the biomarkers required should have clear and standardized detection methods, as well as evidence linking the biomarker to the underlying biological mechanisms and clinically significant treatment outcomes.

Second, clinical outcomes should be aligned with the biological effects of inflammation. Traditional depression rating scales such as the Hamilton Depression Rating Scale can reflect the overall severity of symptoms, but may lack sensitivity to the specific neurobehavioral consequences caused by inflammatory disorders [199]. Future trials should therefore incorporate validated assessments of symptom dimensions that may be more closely related to inflammation. Such outcome measures should be well defined, reliable, and clinically meaningful.

Finally, trials should determine whether the intervention has adequately engaged its intended biological target. Longitudinal measurement of proximal endpoints, such as changes in CRP, cytokines, soluble cytokine receptors, or cerebrospinal fluid markers, can help distinguish failure of the therapeutic hypothesis from inadequate modulation of the target pathway.

## 7. Limitations and Future Directions

A major limitation in the current literature is the uncertain relationship between peripheral inflammatory markers and central neuroinflammatory processes. Many studies using CSF measurements or PET-based indices of neuroinflammation have not found consistent correlations between peripheral inflammatory markers and central immune alterations [200]. This indicates that the cytokines present in the circulation should not be regarded as direct indicators of neuroinflammation within the brain. However, this peripheral-central mismatch does not exclude communication between systemic and central immune processes. On the contrary, it indicates that this relationship is likely shaped by several moderating factors, including blood–brain barrier permeability, regional specificity of neuroinflammatory responses and methodological differences across studies [200]. Therefore, future research should combine peripheral biomarkers with CSF measures, neuroimaging markers, assessments of BBB integrity, and longitudinal immune profiling to better define the relationship between systemic inflammation and central neuroinflammation in MDD.

Relevant literature also highlights the significant heterogeneity and inconsistency in the research. For instance, the levels of inflammatory cytokines vary greatly among individuals, and neuroinflammation is not a universal feature of depression. Although inflammation has been implicated in depression, elevated inflammatory markers are observed only in a subset of patients, whereas others show no obvious evidence of inflammatory activation [201]. Likewise, while neuroinflammation may initially arise in the context of systemic inflammatory conditions such as cardiovascular disease, type 2 diabetes, or obesity, not all individuals with these disorders develop major depressive disorder [202,203]. Elevated pro-inflammatory markers, including IL-6, TNF-α, and CRP, have been reported across several psychiatric conditions, such as depressive disorder, first-episode psychosis, and generalized anxiety disorder [204,205]. This indicates that peripheral inflammation may reflect a broader dimension of immune dysregulation in mental disorders. Therefore, intervention measures targeting the immune system may have limited effectiveness in patient groups without immune stratification.

Although immune-based stratification may help identify potential inflammatory subtypes, this approach still has important methodological limitations. Previous meta-analyses have shown that the variability in some inflammatory biomarkers is not significantly greater in patients than in healthy controls, suggesting that a single biomarker may be insufficient to reliably define inflammatory subgroups [122,206]. Future studies should therefore incorporate rigorous control designs, symptom-dimensional analyses, and longitudinal biomarker assessments to determine whether these inflammatory features have genuine diagnostic, prognostic, or treatment-guiding value.

## 8. Conclusions

This review highlights the complex relationship between neuroinflammation and depression, emphasizing the interactions among immune dysregulation, stress-system dysfunction, neurotransmitter imbalance, and impaired neuroplasticity. Neuroinflammatory processes may contribute to depressive symptoms through interconnected mechanisms, including microglial activation, HPA-axis dysregulation, kynurenine pathway alterations, and cytokine-driven impairment of neurogenesis and synaptic plasticity. These mechanisms provide a broader biological framework for understanding inflammatory contributions to depression, particularly in biologically defined subgroups.

Inflammatory biomarkers such as CRP, IL-1β, IL-6, TNF-α, TGF-β1 and BDNF may support patient stratification, treatment monitoring, and assessment of target engagement. However, these biomarkers are unlikely to be universally applicable and should not be interpreted as direct measures of central neuroinflammation. Their main value may lie in identifying patients with elevated inflammatory activity who are more likely to benefit from biomarker-guided therapeutic strategies. Future research should focus on defining reproducible inflammatory phenotypes, clarifying peripheral–central immune relationships, and conducting prospective trials of individualized anti-inflammatory interventions in depression.

## Figures and Tables

**Table 1:** Inflammatory biomarkers and candidate therapeutic targets for depression.

Inflammatory Biomarker	Brief Effect on Neuroinflammatory Process	Detection Location	Values Detected in Depression	Emerging Therapeutics
C-reactive protein (CRP) [17]	CRP is an acute-phase reactant that can reflect the systemic inflammation driven by IL-6.	Plasma	↑	TNF antagonism
Interleukin-6 (IL-6) [118]	Activate the IL-6R/JAK-STAT pathway, thereby influencing the transmission of inflammatory information at both the peripheral and central levels.	Plasma, CSF	↑	IL-6R blockade
Tumor necrosis factor-α (TNF-α) [119]	Activate the NF-κB pathway, thereby altering the function of the blood–brain barrier and synaptic signal transduction.	Plasma, CSF	↑	TNF antagonism
Interleukin-1β (IL-1β) [120]	A mature cytokine activated by inflammasomes can enhance the activity of microglia and interfere with excitatory neural transmission.	Plasma, CSF	↑	NLRP3/IL-1 axis inhibition (NLRP3 inhibitor MCC950)
Interleukin-2 (IL-2) [121]	Adaptive immune activation	Plasma	↑	COX-2 inhibition
Interleukin-12 (IL-12) [122]	Promotes Th1 polarization and cellular immunity, contributing to a type-1 inflammatory environment	Plasma	↑	IL-12/23 p40 blockade
Interleukin-17A (IL-17A) [67]	Recruit myeloid cells and potentiate inflammatory signaling networks.	Plasma	↑	IL-23/Th17 combination therapy drug
Interferon-γ (IFN-γ) [123]	Coordinate antigen-presentation programs and modulate inflammatory metabolism pathways.	Plasma, CSF	≈	Anti-inflammatory adjunct (celecoxib)
Interleukin-23 (IL-23) [124]	Supports Th17 maintenance and may facilitate CNS immune cell recruitment via IL-23/Th17 axis activity.	Plasma	≈	IL-23 p19 blockade (risankizumab)
CCL2 [125]	Promotes the aggregation of monocytes and has the potential to enhance peripheral–central immune transmission.	Plasma, CSF	↑	Anti-inflammatory adjunct (celecoxib)
CXCL10 [126]	IFN-inducible chemokine	Plasma, CSF	≈	Anti-inflammatory adjunct (celecoxib)
NLRP3 inflammasome [127]	Trigger an amplification effect of neuroinflammation in the downstream area.	monocytes	↑	Inflammasome inhibition
Kynurenine (kyn) [128]	Limit the rate of tryptophan decomposition. Link the inflammatory signal transduction to the availability of the neurotransmitter substrate.	Plasma, CSF	≈	Anti-cytokine strategy (infliximab)
COX-2 [129]	Regulate the amplification of inflammation and the signal transduction of glial cells through the production of prostaglandins.	Plasma, CSF	≈	COX-2 inhibition (celecoxib)

Note: ↑ indicates increased levels detected in depression compared with healthy controls; ≈ indicates no consistent or statistically significant change reported across studies. CSF, cerebrospinal fluid.

## Data Availability

No new data were generated or analyzed in this study.
